# The interfacial nature of proximity-induced magnetism and the Dzyaloshinskii-Moriya interaction at the Pt/Co interface

**DOI:** 10.1038/s41598-017-17137-z

**Published:** 2017-12-04

**Authors:** R. M. Rowan-Robinson, A. A. Stashkevich, Y. Roussigné, M. Belmeguenai, S.-M. Chérif, A. Thiaville, T. P. A. Hase, A. T. Hindmarch, D. Atkinson

**Affiliations:** 1 Department of Physics, University of Durham, Durham, DH1 3LE United Kingdom; 2 Laboratoire des sciences des procédés et des matériaux, CNRS UPR 3407, Universitè Paris 13, Sorbonne Paris Citè, 93430 Villetaneuse, France; 3 Laboratoire de Physique des Solides, CNRS UMR 8502, Universitè Paris-Sud, 91405 Orsay Cedex, France; 4 Department of Physics, University of Warwick, Coventry, CV4 7AL United Kingdom; 5 Present Address: Department of Physics and Astronomy, Uppsala University, Box 516 751 20 Uppsala, Sweden

## Abstract

The Dzyaloshinskii-Moriya interaction has been shown to stabilise Nèel domain walls in magnetic thin films, allowing high domain wall velocities driven by spin current effects. The interfacial Dzyaloshinskii-Moriya interaction (IDMI) occurs at the interface between ferromagnetic and heavy metal layers with strong spin-orbit coupling, but details of the interaction remain to be understood and the role of proximity induced magnetism (PIM) in the heavy metal is unknown. Here IDMI and PIM are reported in Pt determined as a function of Au and Ir spacer layers in Pt/Co/Au,Ir/Pt. Both interactions are found to be sensitive to sub-nanometre changes in the spacer thickness, correlating over sub-monolayer spacer thicknesses, but not for thicker spacers where IDMI continues to change even after PIM is lost.

## Introduction

Manipulating magnetisation with current is an extremely appealing prospect for magnetic memory and logic. First realized with spin-polarised current in spin-valves via spin-transfer torque^1^, progress has been limited by the high current densities required to induce domain wall motion^2^. However, more recently the focus has moved towards spin-orbit torques, where the switching is driven by spin-current and leads to more efficient magnetisation control. Such torques are commonly attributed to the Rashba^3,4^ effect or spin-Hall effect^5,6^ at the interface between the heavy metal and ferromagnetic layers.

Extremely high domain wall velocities have been observed in perpendicularly magnetised Pt/ferromagnetic films^7–10^ and it has been suggested that these domain walls are driven by spin-current generated within the Pt and pumped across the interface into the ferromagnet via the spin-Hall effect. It has become clear that the primary spin-orbit torque associated with the spin-Hall effect is a damping-like torque with the same symmetry as the Slonczewski spin-transfer torque between layers in a stack under perpendicular current^11^. However the spin arrangement of the magnetostatically favoured Bloch walls in these systems is such that the damping-like torque is zero and the observed high domain wall velocities therefore require a Néel-type domain wall, which can be obtained in the presence of an interfacial Dzyaloshinskii-Moriya interaction (IDMI)^12,13^ across the interface. The IDMI stabilises a Néel type wall and imposes a chirality upon it, causing successive domain walls to move in the same direction under the spin-orbit torque^14^. The IDMI interaction (anti-symmetric exchange) is given by −***D***·***S***_1_ × ***S***_2_ and favours orthogonal alignment of spins ***S***_1_ and ***S***_2_ at the interface, which can be represented as an effective field acting across the wall that stabilises the chiral Néel configuration. This effect also plays a crucial role in stabilising Skyrmion phases in magnetic thin films^15–18^.

In Pt/ferromagnet heterostructures, there also arises another phenomenon: a spontaneous magnetic polarisation in the interfacial region^19^. This proximity induced magnetism (PIM) in Pt is associated with the large Stoner factor of heavier *d*-transition elements, with Pd, Ir and W all having been shown to exhibit some degree of induced moment when placed in proximity to a ferromagnet^20–23^.

To date, most experiments have focused on understanding the above spin-orbit phenomena^24,25^, and neglected the polarisation of the heavy metal layer. Attempts to distinguish between the Rashba and spin Hall components of spin-orbit torques have indicated an anomalous interfacial contribution^26–28^, and also that the structure, as well as the spin-transparency, of the Pt/Co interface can dramatically modify the spin-orbit torque efficiency or the effective spin-Hall angle^29,30^. However, the specific role of PIM on the magnetisation dynamics and the relationship to IDMI has been highlighted by recent work, where high domain wall velocities were only observed when heavy metal layers thought to exhibit PIM were used^31^. Following this, ultrathin heavy metal spacer layers (SLs), notably Au, which exhibits an extremely weak PIM, were inserted at the Pt/ferromagnet interface, leading to evolutions of PIM, IDMI and domain mobility with SL thickness. These studies suggest there is an intimate link between IDMI and PIM^32^. In contrast, a recent theoretical work found no direct correlation between IDMI and PIM in Pt, at the Co/Pt interface^33^. To gain further physical insights into any possible relationship between IDMI and PIM, and to address the conflict between previous experiments^32^ and theory^33^, requires direct measurements of both the IDMI and PIM in the same samples. Furthermore, the analysis of the magnetic phenomena needs to be considered in the context of the interfacial structure, the details of which are determined by the arrangment and interactions of the atomic consituents across the interface.

Here, experimental analysis is reported for  both the PIM in Pt and the total IDMI in Pt/Co/SL/Pt multilayers, where SL is a spacer layer of Au or Ir, the analysis having been performed as a function of SL thickness. The IDMI and PIM measurements were also correlated using x-ray analysis of the physical structure of the multilayers and their interfaces. The elements for the spacer layer were selected based on previous understanding, where Au was selected following ref.^31,32^ as it is expected to have a negligible PIM^34,35^, and no DMI has been reported^31,32^. In contrast, it has been suggested that Ir takes on a moderate PIM^23^ and a non-negligible IDMI constant, as was recently reported for a Co/Ir structure measured by Brillouin spectroscopy^36^. The difference in PIM is not surprising, considering that the 5d band is full for the noble metal Au, but not for Ir. For IDMI, model calculations specifically aimed at studying the band filling effect have lead to the same conclusion^37^.

Multilayers of Pt(54)/Co(25)/SL(0-25)/Pt(26) (thicknesses in Å units, with the convention that structures are described starting from the substrate) were sputter-deposited, with a spacer layer of either Au or Ir. The cobalt thickness was selected to produce in-plane magnetisation, which was confirmed by the magnetic hysteresis measured using MOKE. In-plane magnetisation enables both PIM measurements and IDMI analysis on the same samples. The relatively large cobalt thickness is also useful for having well-separated interfaces.

### Interfacial Dzyaloshinskii-Moriya interaction as a function of Au and Ir spacer layers

**Figure 1:**
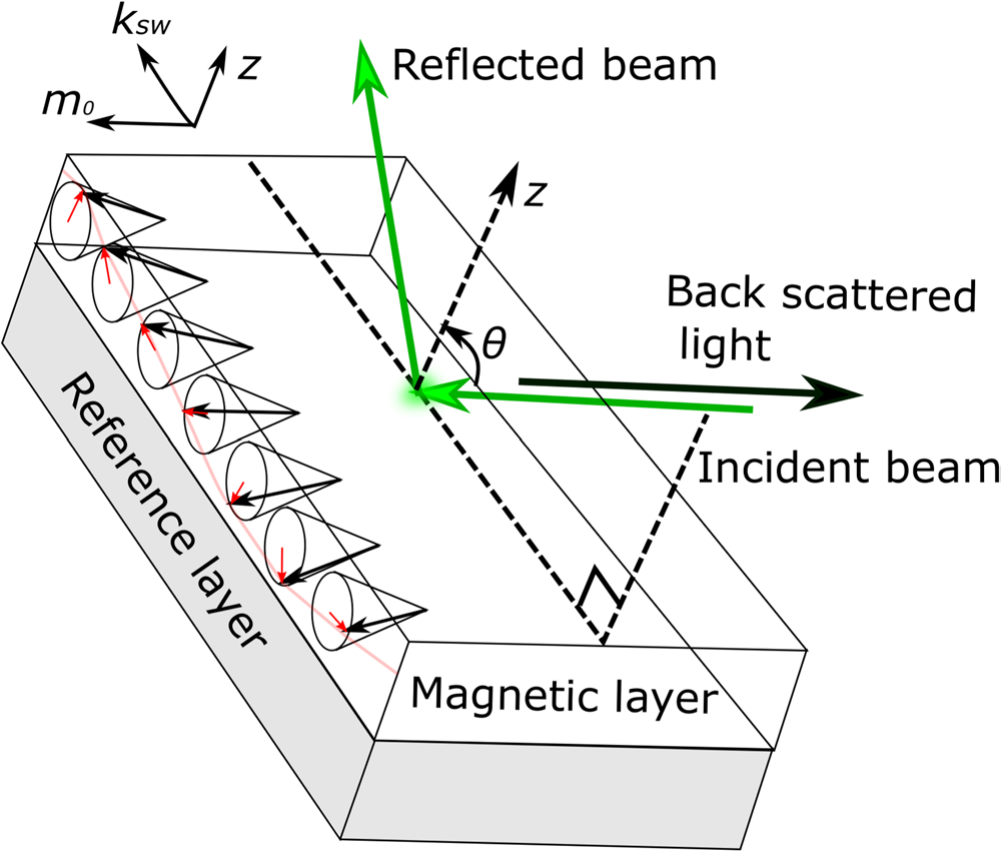
Schematic for the chirality of spin waves inside a magnetic film in the Damon-Eshbach geometry. One first has to define the oriented film normal (*z*), from a chosen reference layer to the film (usually the reference layer is the heavy metal whose spin-orbit interaction gives rise to IDMI). The wave-vector of the spin wave is *k*_*sw*_, and the static magnetisation is *m*_0_. A snapshot of the precessing moment is shown, for the depicted orientations of the vectors *k*_*sw*_ and *m*_0_. It corresponds to an anti-clockwise cycloid. Such is the case when (*k*_*sw*_, *m*_0_, *z*) is a right-handed frame. For the light incidence configuration shown, such a spin wave would be measured in the Stokes configuration. Finally, the convention^70^ for the IDMI sign is that a positive IDMI favors clockwise cycloids, i.e.it decreases energy and frequency of such spin waves.

The IDMI was measured by Brillouin light scattering (BLS)^38^, which detects light inelastically scattered by excitations. BLS probes the dispersion relation of these excitations, in this case thermally populated magnons, through momentum (wave number) and energy (frequency) resolution. The influence of IDMI on the spin wave (SW) spectrum is now well-known, both theoretically^39–42^ and experimentally^43–46^. IDMI induces a characteristic non-reciprocity of the SW propagation, such that SWs with the same wave-number, but travelling in opposite directions have different frequencies. The symmetry of IDMI implies that the SWs must be probed in the so-called Damon-Eshbach (DE) geometry^47^, in which the magnetisation and the wave-vector are in-plane and mutually perpendicular. In this configuration the frequency shift of the two counter-propagating SW modes is largest, and scales inversely with the sample thickness, which eliminates any contribution to the non-reciprocity due to asymmetric surface magnetic anisotropy^38^. The two counter-propagating modes are recorded within the same spectrum with one as an energy loss (a frequency down-shifted Stokes mode, denoted ‘S’) and the other as an energy gain (a frequency up-shifted anti-Stokes mode, denoted ‘AS’). The experiments were performed in the back-scattering geometry, as shown in Fig. 1. The spectra were obtained by counting photons for typically 12 hours, allowing the mode line positions to be determined with a precision better than 0.1 GHz. The Stokes (*f*_*S*_) and anti-Stokes (*f*_*AS*_) frequencies were determined from Lorentzian fits to the spectral peaks. More detail on the technique and analysis can be found in the methods section.

Previous experimental work in ultrathin films^45,46^ has shown that the effective IDMI is *D*_eff_ = *D*_s_/*t*_FM_, where *t*_FM_ is the ferromagnetic thickness and *D*_s_ is the IDMI surface (or interfacial) parameter. For well-separated interfaces, it is expected that *D*_s_ represents the difference between the IDMI contributions from the bottom and top interfaces. The IDMI then leads to a pure frequency shift of the SWs according to their chirality that results in a frequency shift, Δ*f* ≡ *f*_*S*_ − *f*_*AS*_, which can be converted into the IDMI constant *D*_*s*_ using^45^

1 $${D}_{{\rm{s}}}={D}_{{\rm{eff}}}{t}_{{\rm{FM}}}=\frac{\pi }{2{\rm{\gamma }}}\frac{{M}_{{\rm{s}}}{t}_{{\rm{FM}}}{\rm{\Delta }}f}{{k}_{{\rm{sw}}}},$$

where γ is the gyromagnetic ratio γ = *gμ*_B_/*ħ* = *g* × 8.794 × 10^6^ Hz/G and *k*_sw_ is the SW wavenumber. The SW wavenumber is given by

2 $${k}_{{\rm{sw}}}=\frac{4\pi }{{{\rm{\lambda }}}_{{\rm{opt}}}}{\rm{s}}{\rm{i}}{\rm{n}}(\theta )$$

where *θ* is the angle of incidence and λ_opt_ the wavelength of the incident light (532 nm). The linearity as a function of *k*_sw_ was confirmed and the incidence angle was fixed at *θ* = 50°, giving *k*_sw_ = 19 *μ*m^−1^. For this experimental arrangement and taking values for the saturation magnetisation of Co of *M*_*s*_ = 1400 kA/m and the g-factor of *g* = 2.17, *D*_s_ can be related to Δ*f* by the relation *D*_s_ = 1.59 × Δ*f*, where Δ*f* is in GHz and *D*_*s*_ in pJ/m.

**Figure 2:**
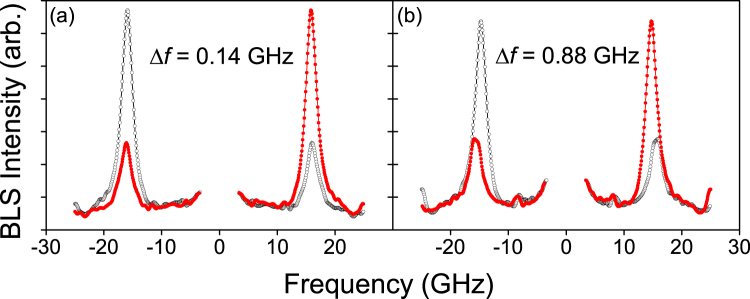
Raw BLS spectra for (**a**) Pt/Co/Au (2 Å)/Pt and (**b**) Pt/Co/Au (16 Å)/Pt samples. The data shown by black (open) markers was obtained with a +2 kOe in-plane field and at *θ* = 50 deg, while the red (filled) markers represent the same spectra but plotted reversed about zero frequency, so as to demonstrate the frequency shift undergone by the magnon peaks.

Representative spectra for very thin (2 Å) and thick (16 Å) Au SLs are shown in Fig. 2. The solid markers in the figure show the original measured spectra reflected on the horizontal (frequency) axis, in order to highlight the frequency shifts. This analysis shows that for the very thin SL, the Stokes and anti-Stokes SW peaks in the direct and the frequency-inverted spectra are almost superposed (*f*_S_ ≈ *f*_AS_), indicating a very small net IDMI, while the spectrum with the thicker Au SL shows a pronounced frequency difference, indicative of a net IDMI contribution. The difference between the Stokes and anti-Stokes frequencies, i.e. *f*_S_ < *f*_AS_ for a positive field, gives information about the sign of the IDMI, which is negative here, i.e. favors left-handed cycloids^45^. A description of this sign convention is given in Fig. 1. The measurement reliability was maximised by measuring the BLS spectra with positive and negative magnetic field polarities to take advantage of the fact that the sign of the frequency shift Δ*f* changes when the polarity of the saturating magnetic field is reversed.

**Figure 3:**
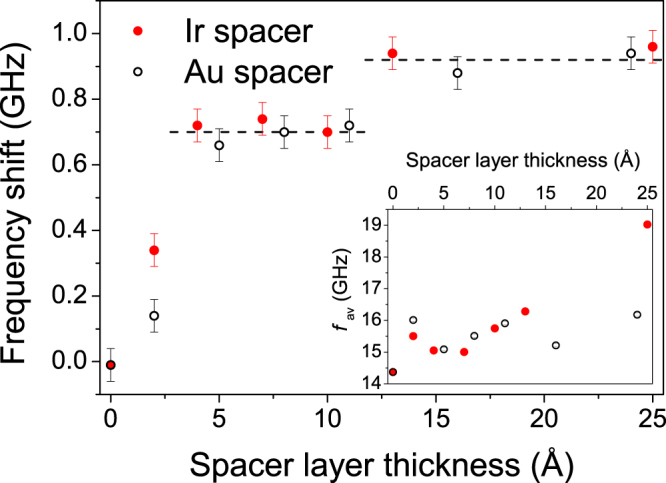
The frequency shift due to the IDMI are plotted against nominal spacer layer thickness for Au spacers (open markers) and Ir spacers (filled markers). The rise in IDMI is due to breaking the symmetry of the Pt/Co/Pt structure, removing the cancellation of the IDMI contributions from the top and bottom Pt interfaces. Dashed lines indicate apparent plateau values of the frequency shift due to IDMI. Inset: Average frequency, *f*_av_ = (*f*_S_ + *f*_AS_)/2, extracted from BLS, as a function of nominal spacer thickness. Error bars in *f*_av_ are smaller than the size of the datapoint markers.

The change of effective IDMI as a function of Au and Ir nominal spacer layer thickness is summarised in Fig. 3, where the absolute value of the IDMI-induced frequency asymmetry |Δ*f* |, termed the frequency shift, is plotted as a function of the nominal SL thickness *t*. The first thing to note is that with no SL, no net effective IDMI was observed. Within measurement resolution this indicates perfect cancellation of the IDMI contributions from the top Co/Pt and bottom Pt/Co interfaces here. This is interesting since the Co on Pt interface typically has better structural quality than the Pt on Co interface^48^. Measurements of IDMI through domain expansion^49^ found that, with the exception of epitaxial structures, the asymmetry of the microstructure between the bottom Pt/Co and the top Co/Pt interfaces gave rise to a net *D*_s_ in a nominally symmetric Pt/Co/Pt multilayer (although the Co was much thinner and the magnetisation out of the plane, compared to the present study). In contrast, good symmetry was observed in BLS measurements^50^ on CoFe/Pt and Pt/CoFe stacks.

The increase in the net effective IDMI observed with increasing SL thickness occurs over a lengthscale that is consistent with the interface width, which is approximately 5 Å, as determined from XRMR analysis (see below), and was similar for both Au and Ir spacer layers. The thickness dependence shows an initial rapid rise with SL thickness followed by an apparent two-step approach to saturation for both Au and Ir spacer layers (based on an observed step height of 200 MHz and inter-channel frequency separation of 82 MHz). Considering the finite interface width, the final step change may be associated with the formation of a continuous spacer layer that completely covers the Co, which is similar to the effect shown recently for the heavy metal capping layer thickness dependence of magnetisation damping of cobalt^51^. Alternatively, the step may reflect variations arising from sampling of oscillatory behaviour superimposed onto a smooth exponential approach to saturation.

Within experimental precision, for both Au and Ir SLs the frequency shifts saturate at −0.95 ± 0.01 GHz. This corresponds to an effective value, *D*_eff_ = −0.60 mJ/m^2^ and an interfacial parameter *D*_s_ = −1.51 pJ/m. Assuming well-separated interfaces, this result can be compared to measurements and calculations on other systems. For the Ir SL, a recent Brillouin spectroscopy study^36^ on Ta/Pt(Ir)/Co/AlOx measured *D*_s_(Ir/Co/AlOx) ≈ −0.9 pJ/m and *D*_s_(Pt/Co/AlOx) ≈ −2.2 pJ/m. Interestingly, for the latter stack without a Ta seed layer the IDMI was smaller ≈ −1.4 pJ/m, while another study of Pt/Co/AlOx^45^ gave an intermediate value of *D*_s_ = −1.7 pJ/m. Together, these results indicate a sensitivity of the IDMI to sample details, probably the interface structure. From the differenceof the first two quoted results^36^, one obtains *D*_s_(Pt/Co/Ir) ≈ −1.3 pJ/m, which is in reasonable agreement with the value obtained in this work, bearing in mind the noted sensitivity of IDMI to the interfacial structure.

Regarding the effects of Ir or Au SLs, *ab initio* calculations^33,52^ give *D*_s_ = 0.67 pJ/m for Au/Co and *D*_s_ = 0.42 pJ/m for Ir/Co interfaces. The difference between these values of *D*_s_ would give a frequency shift of $\frac{2{\rm{\gamma }}{k}_{sw}}{{\rm{\pi }}{M}_{s}{t}_{FM}}$ [*D*_*s*_(Au/Co) −*D*_*s*_(Ir/Co)] =157 MHz, which is just above the experimental detection limit. Taking into account that these values are calculated for ideal systems (for example the same calculations predicts *D*_s_ = −3.41 pJ/m for Pt/Co), the observed similarity of the Δ*f* saturation values for the two SL systems is not a surprise. Finally, considering the sign of *D*_*s*_(Ir/Co), the data in this study does not resolve the conflict between the positive calculated value^33^ and the negative measured value^36^, under the assumption that *D*_*s*_(Co/AlOx) is small, as supported by calculation^17^ and experiment^53^. Indeed, this depends on the IDMI of our bottom Pt/Co interface.

The insert in Fig. 3 shows the average frequencies *f*_av_ = (*f*_S_ + *f*_AS_)/2 as a function of SL thickness *t*. First, recalling that at *k*_*sw*_ = 0 the resonant frequency is represented by the Kittel formula $f=({\rm{\gamma }}{\mu }_{0}/2\pi )\sqrt{H(H-{H}_{K}+{M}_{s})}$, any changes of the interfacial anisotropy may be tracked through changes of the effective anisotropy field, *H*_*K*_, as a function of the SL thickness. For the Au SL, only small scale variations were observed, which, at first sight, is consistent with the fact that Au/Co and Pt/Co have similar interfacial anisotropy constants^54^, however the measurement uncertainties are smaller than the scatter of the points suggesting changes of the interface structure from sample to sample. For the Ir SL, similar behaviour was observed, except for the largest thicknesses. These results are surprising since Ir/Co has a larger interfacial anisotropy than Pt/Co^54^ so a decrease in the frequency was expected.

**Figure 4:**
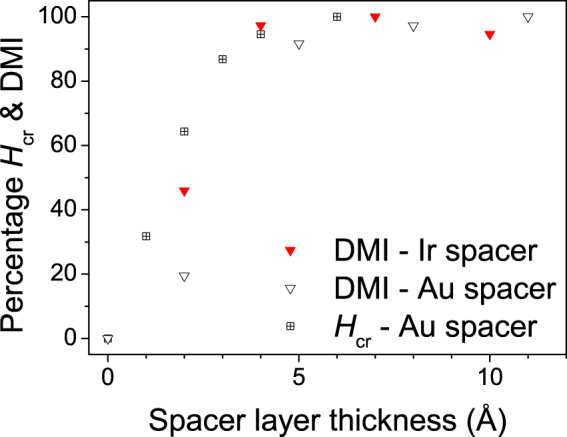
Comparison of crossing field, *H*_cr_, from ref.^31^ and experimental values of effective IDMI with Au and Ir SLs from this work, across the range of comparable SL thicknesses available. *H*_cr_ is normalised to the maximum value, and IDMI is normalised to the first plateau value, relevant to that thickness range.

The IDMI constants obtained here are compared with the results from high-velocity domain wall (DW) dynamics^31,32^. The structure of the films used in those studies was similar, Pt(50 or 15)/Co(3)/Ni(7)/Co(1.5)/TaN(50), with the caveat that instead of a 25 Å Co film,a Co/Ni/Co trilayer was used. In the domain wall dynamics studies, a longitudinal magnetic field was applied to cancel the effective field at the DW due to the IDMI. A “crossing field” was defined as proportional to the effective IDMI such that *H*_cr_ = *D*_eff_/(*μ*_0_ *M*_*s*_Δ), where Δ is the DW width parameter. The crossing field was observed to decrease for sub-nanometric Au spacer layers inserted at the lower Pt/Co interface^31^. In Fig. 4 the IDMI variation (expressed as a percentage of the first plateau value) obtained here is compared to the crossing fields from Ryu *et al*., where the reduction in the crossing field value is also presented as an increasing percentage, such that the measured reduction of the crossing field appears as an increase. A similar variation is observed as a function of spacer layer thickness for both methods on all curves, for the available SL thicknesses up to 6 Å. Thus, estimates of the IDMI strength as a function of SL thickness from two completely different experimental techniques correlate well over the range of SL thicknesses available. However, it is noted that the DMI reported here exhibits an additional step increase for the thickest spacer layers, which is a non-trivial result since there exists a pronounced discrepancy between the *H*_cr_ measured for different heavy metal underlayers in the above cited papers^31,32^ and the values for the IDMI constants extracted from more recent independent BLS experiments^36^. For example, Ryu *et al*. report an eight fold difference in the value of the *H*_cr_ in Pt/CoNiCo and Ir/CoNiCo structures whilst the BLS measurements reported in ref.^36^ indicate a difference that does not exceed three times.

### Proximity induced magnetism as a function of Au and Ir spacer layers

**Figure 5:**
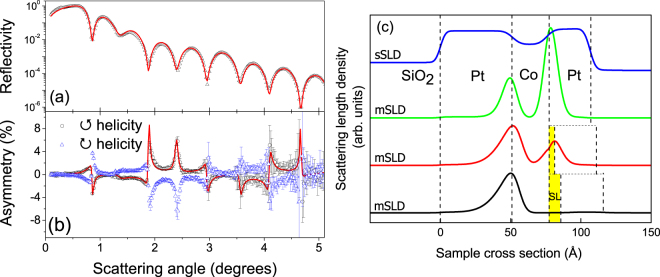
(**a**) Example of specular reflectivity data (markers) for no spacer layer and the best fitting simulation (line). (**b**) Spin asymmetry data for the same sample (markers) and best fitting simulation (line). (**c**) Scattering length density profiles extracted from the best fit simulations. Upper curve structural profile (sSLD) corresponding to the sample with no SL, second curve magnetic profile (mSLD) with no spacer layer, and lower curves mSLD with 5 Å and 10 Å of Au, respectively. The peaks in the mSLD coincide with the Pt interfaces (vertical dashed lines) and the area under them is related to the total moment induced at the interface.

Proximity induced magnetism in Pt was extracted using x-ray resonant magnetic reflectivity (XRMR)^55,56^, which is an element-specific technique and therefore sensitive only to the Pt moment when performed using circularly polarised x-rays tuned to the energy of the Pt *L*_3_ absorption edge. The primary experimental quantity related to PIM from the XRMR measurements is the spin asymmetry ratio (*R*_a_), defined as *R*_a_ = (*I* ^+^ − *I* ^−^)/(*I* ^+^ + *I* ^−^) where *I* ^+^ is the scattered intensity of the circularly polarized x-rays when the sample is magnetised with a positive in-plane magnetic field and *I* ^−^ is the equivalent with negative magnetic field (sign conventions are the same as in Fig. 1). The reflectivity geometry allows the depth dependence of the PIM to be extracted from simultaneous best fitting simulations of the measured specular reflectivity, *R*_s_ = (*I* ^+^ + *I* ^−^)/2, (Fig. 5(a)) and the spin asymmetry ratio (Fig. 5(b)), thereby constraining the best fitting magnetisation profile to be consistent with the physical structure of the sample. For the simulations the sample was modelled as slabs of Pt, Co and a SL of various thickness and interfacial roughness. From the model, the structural scattering length density profile (sSLD) was extracted, an example of which is shown in the upper trace of Fig. 5(c). The spin asymmetry ratio was fitted by modelling the sample structure with a distribution of magnetic moments through the thickness of the Pt, which gave the magnetic scattering length density profile (mSLD), examples of which are shown in the lower traces of Fig. 5(c). More detail on the technique and analysis can be found in the methods section.

Figure 5(c) shows the sSLD that represents the structure of the Pt/Co/Pt sample. The plateaus correspond to the bottom Pt and top Pt layers separated by the lower (non-resonant) sSLD of the Co and the slopes in-between indicate the interface widths. The corresponding mSLD for Pt/Co/Pt is shown immediately below the sSLD. A peak occurs at each Pt/Co interface, indicated by the vertical dashed lines. The area under each peak is proportional to the total induced Pt moment at that Pt interface. The lower Pt/Co interface is the same for all samples (nominally at least), so the induced moment can be assumed constant, such that for samples with increasing SL thickness, any variations of experimental conditions between samples is accounted for by normalizing the area under the Co/SL/Pt peak to the area under the lower Pt/Co layer mSLD peak, as a function of SL. Let us now consider the details of the induced Pt moments at the upper and lower interfaces. We first note that the induced Pt moment for the sample with no SL shows a larger moment for the top Co/Pt interface compared to the lower Pt/Co interface. Such asymmetries of the induced moment have also recently been observed in Pd/Co/Pd structures^57^, suggesting differences in the interfacial structures at the top and bottom interfaces of the Co layer that result from the growth of Co onto Pt and Pt onto Co respectively. A tomographic atomic probe study of Pt/Co multilayers^48^ did similarly find a difference in the composition profile across the Pt/Co interface relative to the Co/Pt interface. However, no evidence of different interfacial structures (i.e. interface roughness) was observed in the best fits to the specular reflectivity, which may reflect the subtlety of any differences in interfacial structure. Nonetheless, the result suggests that the proximity induced magnetisation of Pt is very sensitive to the local atomic structure and associated electronic interactions.

Interestingly, the difference in induced moment between the top and bottom Pt interfaces is not reflected in the IDMI measurement, which within the measurement resolution showed perfect cancellation of the IDMI contributions. Since the IDMI is also sensitive to the structure at the interface, the different sensitivities to the interface structure of PIM and the IDMI argue against the direct linkage between PIM and IDMI^32^. Note also that, if the difference in the Pt moments induced at the bottom and top interfaces was due to different degrees of interfacial intermixing, not resolved by the specular reflectivity measurements, this would contradict the *ab initio* calculations of Yang *et al*.^33^ that suggested intermixing has a large detrimental effect on the IDMI. Thus, it may be concluded that other differences in interfacial structure may contribute to the differences in the proximity induced moments.

With the addition of a sub-nanometric SL, the mSLD changes significantly, with the moment on the top Pt layer falling dramatically for both Au and Ir spacer layers. As shown in Fig. 5(c), the insertion of a nominal 5 Å layer of Au significantly changes the magnetic profile of the top Pt layer, reducing the induced Pt moment at the top interface to below that of the bottom interface. This further demonstrates the exquisite sensitivity of the PIM to the structure of the interface, in particular to the immediate proximity of Pt to Co. The addition of 10 Å of Au between the layers almost entirely destroys the induced magnetism in the upper Pt layer.

**Figure 6:**
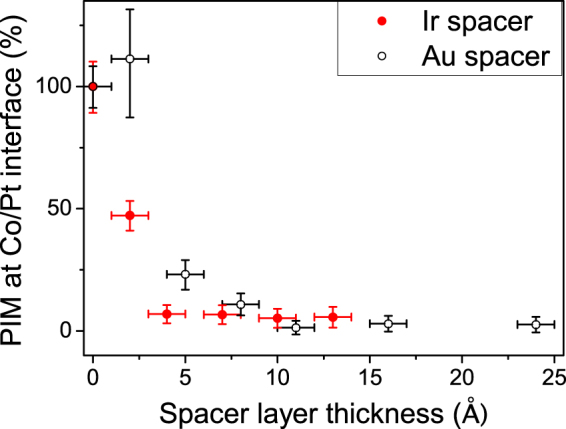
Percentage of Pt moment at top interface as a function of Au or Ir nominal spacer layer thickness. The Pt moment falls rapidly with increasing spacer layer thickness.

The PIM as a function of Au and Ir spacers is summarised in Fig. 6, where the ratio of the areas under mSLD peaks of the upper Pt and lower (fixed) Pt is plotted as a function of the SL thickness, normalised such that the sample with no spacer layer has a value of 100%. For the Ir spacer the PIM in the Pt layer is completely lost (within experimental error) for a SL thickness of 4–7 Å, whereas with Au SL the Pt PIM is lost with a SL thickness of 11 Å. Thus Ir seems to cause the PIM at the top Co/Pt interface to reduce more rapidly, while the Au thickness dependence is a little more gradual. The indication that Ir causes a more rapid loss of the Pt PIM may be considered surprising since Ir can take on a proximity induced moment^22,23^, which may have been considered to mediate any polarising interaction between Co and Pt.

When considering the role of ultrathin SLs the nature of the interface is important. From analysis of the specular x-ray reflectivity the interface width of the Co/Pt interface was determined to be of order 5 Å, which can be considered simplistically as a guide to the minimum SL thickness required to create a continuous layer at the interface. The difference between Au and Ir is their ability to alloy with Co and Pt. Au is almost immiscible with Pt^58^ and Co, so sub-nanometric SLs of Au would form discontinuous layers allowing a significant amount of direct contact between Co and Pt at the interface. In contrast, Ir alloys well with Pt^59^ and Co, so an Ir SL may locally intermix with Pt and Co, which would cause significant modification to the density of states of Pt and Co. The interfacial Pt may then become less strongly proximity polarised as the Stoner enhanced paramagnetic susceptibility would be reduced from that of pure Pt (the molar susceptibilities are 201.9 and 25.6, in units of 10^−6^ cgs/mol, for Pt and Ir, respectively). For ultrathin Au SL the coverage would lead to a greater roughness with minimal intermixing at the SL/Pt interface, compared to a more intermixed interface with lower roughness for the Ir SL. These structural interface components cannot be resolved using specular reflectivity. So, the PIM dependence on the nominal SL thickness can also be related to the interfacial structure; where the local interfacial Co-Ir-Pt alloying leads to a more rapid loss of PIM with nominal SL thickness than does the more gradual reduction in direct Co/Pt contact caused by the insertion of a sub-monolayer Au SL.

### Relationship between IDMI and Proximity induced magnetisation in Pt

The characteristic features can be summed up briefly as follows: both IDMI and PIM demonstrate a rapid approach to saturation as a function of SL thickness, but they do not follow the same trend. The PIM falls rapidly with both Au and Ir SLs. For Ir, a nominal thickness greater between 4 and 7 Å is required for the complete loss of the Pt moment, which coincides with the thickness required for complete sign reversal of the IDMI-generated in-plane magnetic field, *H*_DMI_, observed for perpendicular Pt/Co/Ir/Pt multilayers in ref.^49^. The IDMI rises rapidly with the initial insertion of a SL, but the approach to saturation with thicker SLs occurs via a possible two step process, which is not reflected in the trend of the PIM data. The 2 Å Au SL sample deserves a special comment. As shown by the large error bars on its PIM, the XRMR data could not be fitted as well as for the other samples. The specificity of this sample is also apparent on the frequency shift data (Figs 3 and 4) and even maybe on the resonance frequency (Fig. 3, inset). Thus, this data point should be considered with some care.

**Figure 7:**
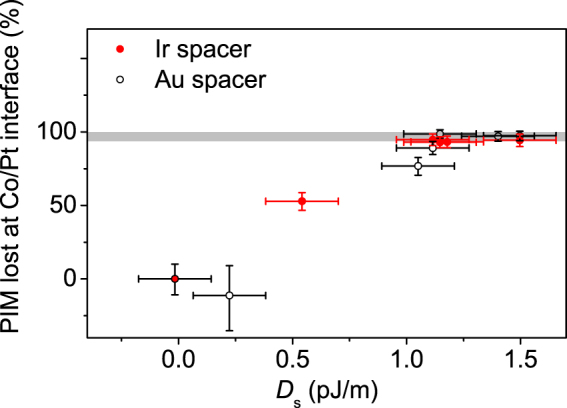
The percentage proximity induced moment lost at the top Co/Pt interface plotted against the *D*_s_ for Au and Ir SLs, which displays an apparent linear correlation at low thickness. The grey bar denotes the region where the PIM is considered to have vanished, based on the sensitivity of the XRMR measurement.

To further investigate potential correlations between the IDMI and PIM, in Fig. 7 the percentage of the Pt proximity moment lost from the top interface is plotted against the effective net IDMI constant, *D*_s_. For the thinnest nominal SL thickness the trend is linear, although the number of data points in the small IDMI region is somewhat limited. Subsequent data for the thickest spacer layers indicate that the PIM is lost more quickly than the IDMI changes, as a function of increasing SL thickness. The data also show that the IDMI continues to rise as the SL thickness increases for thicknesses where the induced moment is completely absent.

The PIM is related to the density of states, while the IDMI is directly linked to the spin-orbit interaction (SOI). Theory has so far proposed several mechanisms of the anisotropic exchange interaction. While Dzyaloshinskii^12^ was the first to predict it from purely symmetry grounds, it was Moriya^13^ who suggested that in low symmetry dielectrics it can be seen as the combined effect of the SOI and exchange interaction. His formalism is essentially an extension of the superexchange theory to include the effect of SOI, in other words the key role is played by the “*d*-*d*” hybridization of core electrons (*d*-*d* exchange mechanism). A completely different mechanism was put forward by Fert and Levy^60^ to explain a peculiar magnetic behaviour of spin glasses with nonmagnetic heavy-metal impurities. The experimentally observed enhancement of the anisotropy field in spin glasses was proposed to arise from an additional term in the Ruderman-Kittel-Kasuya-Yosida (RKKY) interaction of the Dzyaloshinskii-Moriya type, due to spin-orbit scattering of the conduction electrons by the nonmagnetic impurities, which implies an active role played by itinerant “*s*” electrons (*s*-*d* exchange mechanism). This mechanism may be more relevant to the metallic structures investigated in this study. A signature of the conduction electron dominated RKKY interaction is an oscillatory behaviour as a function of spacer thickness *t* with a characteristic period ${\rm{\Lambda }}=\frac{{\lambda }_{F}}{2}$, where *λ*_*F*_ is the Fermi wavelength^61^. In Au, *λ*_*F*_ is on the order of 5 Å, thus Λ ≈ 2.5 Å, which makes such short-period oscillations practically undetectable experimentally. What can actually be observed in an experiment are multiperiodic long-range (≈10 Å) oscillations^62–64^ that occur due to the discrete character of the atomic structure and a beat phenomenon arising from the incommensurability of *t* and inter-atomic distance^65^. In this regard, the step-like irregularities featured by the DMI dependence on SL thickness curve bear a resemblance with the ripple produced by RKKY conduction electron interferences, and thus may attest to the relevance of itinerant electrons to the IDMI mechanism. Note that the oscillatory nature of the RKKY-based IDMI mechanism in metals appears directly in the description proposed by Fert^60^. No such features occur in the PIM SL thickness dependences, which is not surprising since the RKKY mechanism is not associated with the PIM. In other words, the differences in the evolution of the IDMI and PIM with increasing spacer thickness suggest a predominant role played by the itinerant electrons in the case of IDMI, compared to bound *d*-electrons in the case of PIM. Note finally that the results reported here are reminiscent of the slow Pt thickness dependence of IDMI recently reported^66^.

## Conclusions

PIM and the effective IDMI at the Co/Pt interface have been investigated in detail as a function of spacer layers of Au and Ir of various thickness. The SL thickness dependence of both IDMI and PIM demonstrate a rapid change as a function of SL thickness, but not in the same way. First, there exists a major difference in the nominally symmetric Pt/Co/Pt trilayer without a SL. In this structure the proximity induced magnetism manifests a pronounced asymmetry, the PIM developed within the top Pt layer being at least twice that of the bottom one. In contrast, the net effective IDMI in the same Pt/Co/Pt system is characterized by a symmetric spatial pattern, with identical IDMI contributions from the Pt/Co and Co/Pt interfaces leading to a complete cancellation of both contributions, i.e. a zero net IDMI effect. Secondly, for both Au and Ir SLs the PIM decays rapidly in a regular monotonic fashion, vanishing for SL thickness greater than about 10 Å. For Ir the decay is slightly more rapid, with all PIM destroyed by SL thickness of 4–7 Å, a feature that has been ascribed to the capacity of Ir to alloy with Pt and Co, contrarily to Au. The observed increase of the effective IDMI with SL thickness occurs on a lengthscale that is consistent with the interfacial width, as determined by XRMR, and the lengthscale is similar for either Au and Ir spacer layers. For both Au and Ir spacer layers the net IDMI saturates to the same value of *D*_s_ = −1.51 pJ/m, in reasonable agreement with other experiments and calculations. Moreover, the approach to saturation takes place in a characteristic two-step manner. This variation may be associated with the formation of a spacer with a complete coverage of the Co. Alternatively, this feature bears a resemblance with the oscillation produced by RKKY conduction electron interferences, cautiously suggesting the relevance of the itinerant *s*-electrons to the IDMI mechanism.

## Methods

### Film deposition

Thin film multilayers were deposited by DC magnetron sputtering at ambient temperature in an ultra-high vacuum deposition system with base pressure better that 1 × 10^−8^ Torr. Argon working gas pressure is typically 1 mTorr and DC discharge powers are around 25 W, giving deposition rates in the region of 0.25 Å/s. Films are deposited onto commercial Si(001) wafer substrates with 1000 Å thermally-grown amorphous oxide surface coating.

### Analysis of X-ray resonant magnetic reflectivity

When simulating the sample, to accommodate the thin spacer layers the sample is sliced into 0.5 Å sections. A scattering length density is calculated for each of these sections. This is calculated as the product of the fitted electron density for that particular slice and the x-ray scattering factor for the material, in units of Thompson scattering lengths per cubic angstrom.

The x-ray scattering factors can be written as^67^,

3 $$f(Q,E)=({\hat{\varepsilon }}_{f}\cdot {\hat{\varepsilon }}_{i}){F}^{0}(E)-i({\hat{\varepsilon }}_{f}\times {\hat{\varepsilon }}_{i})\cdot \hat{m}{F}^{1}(E).$$

Here, *Q* and *E* are the scattering vector and x-ray energy respectively. ${\hat{\varepsilon }}_{{\rm{f}}}$ and $\hat{\varepsilon }$ are unit vectors describing the polarisation state of the incident and scattered x-rays. *F* ^0^ is the charge scattering amplitude, comprised of both real *f*_0_ + *f* ′(*E*) and imaginary *f* ′′(*E*). Where *f*_0_ is the Thompson scattering factor, roughly equal to the atomic number of the element with *f* ′ and *f* ′′ the anomalous dispersive corrections due to the energy dependence cloe to resonance. Finally, *F* ^1^ is the equivalent magnetic scattering amplitude, containing analogous real *m*′ and imaginary *m*′′ components which account for the magnetisation and polarisation dependence.

The separate slices are then arranged alongside each other to produce a profile through the sample. This profile is then used to calculate the Fresnel coefficients necessary for the Parratt recursive method employed by the model. The parameters are then fitted using a differential evolution algorithm^68^ which adjusts the SLD profiles until a good fit is found for the data.

Due to the large number of parameters used to fit a given model, it is essential to constrain the fit. We have achieved this by simultaneously fitting the reflectivity alongside the spin asymmetry, thereby ensuring all the structural parameters are consistent with both datasets. Furthermore the resonant scattering corrections, *f* ′ and *f* ′′ as well as the magnetic scattering factors *m*′ and *m*′′, which describe the resonant behaviour of the Pt, were determined from the sample with no SL and fixed for the other samples in the series. This approach allows for direct comparisons to be made between the samples, but means an absolute magnetic moment can not be established. However, it is the relative change in magnetism as a function of SL that we are interested in.

### Experimental procedure and analysis of BLS spectra

**Figure 8:**
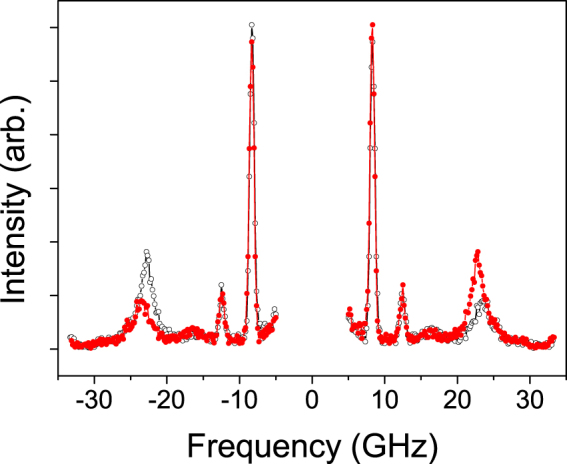
Measured BLS spectrum obtained in the absence of the analyser. Data shown by black (open) markers are obtained at +4 kOe in-plane field and at *θ* = 50 deg. Data in red (filled) markers show the +4 kOe field spectrum reflected.

To ensure reliable Stokes/anti-Stokes frequency asymmetry detection, both the experimental procedure and processing of BLS spectra have been arranged accordingly. On the experimental level the main risk lies with the zero frequency shift in the BLS spectra. This is especially important in the case of ultrathin SLs, where the net IDMI tends to zero through the mutual cancellation of the two contributions from opposite interfaces and, consequently, the Stokes/anti-Stokes frequency asymmetry is very small. Thus, to minimize the instrumental error the following complementary measurements have been taken. First, we were periodically taking BLS spectra without an analyser, thus recording not only the peaks corresponding to light scattering by magnons, but also those representing phonons. Importantly, the propagation of acoustic phonons is never non-reciprocal, hence Δ*f* ≡ *f*_*S*_ − *f*_*AS*_ = 0 which makes them a very effective instrument for checking the calibration of the frequency sweeping of the BLS set-up. This is illustrated in Fig. 8 which shows a representative spectrum (red solid line), obtained at an incidence angle *θ* = 50° (*k*_*SW*_ = 19 *μ*m^−1^) and without analyser such that the phonon lines could also be represented in the spectrum. The spectrum given by the black dotted line is the BLS distribution reflected in the vertical axis in order to show the frequency shifts more clearly. It contains three peaks for negative (Stokes) and positive (anti-Stokes) frequency shifts. The two low-frequency peaks around 10 GHz that do not shift with field and disappear in the crossed polarisers configuration are produced by phonons while the third high-frequency one at ±23 GHz, whose frequency position is directly controlled with an external magnetic field is due to BLS from magnons. As can be seen in Fig. 8 both phonon spectra are absolutely symmetric, the Stokes and anti-Stokes lines in the direct (solid red) and frequency-inverted (dotted black) spectra are perfectly overlapping (*f*_*S*_ = *f*_*AS*_), whilst the magnon-related line demonstrates a clearly seen asymmetry (*f*_*S*_ < *f*_*AS*_). It was routine in measurements to place the analyser in front of the Fabry-Perot interferometer to improve the signal to noise ratio. Moreover, to double check it, we were taking advantage of the fact that the sign of Δ*f* is changed if the saturating magnetic field polarity is inversed. Spectra were obtained after counting photons for typically 12 hours to improve the quality of the raw data BLS spectra.

Numerical simulations of the shape of the BLS spectra of SW in the DE geometry were very helpful for improving the accuracy and reliability of the measurements. They were performed with the help of an *ad hoc* code based on the fluctuation dissipation theorem to evaluate the thermally activated SW and the Green functions associated with the light scattering^69^. For the analysis contained herein we have introduced appropriate boundary conditions^42^ to calculate spectra in the case of a ferromagnetic film between two layers having different surface anisotropies and IDMI constants. These calculations provide the positions as well as the heights of the SW lines. In particular, they have have permitted estimation of the contribution to Δ*f* of the conventional one-sided surface magnetic anisotropy which has turned out to be under 0.01 GHz, in other words far too small to undermine the reliability of our observations. On the other hand, the above-mentioned code has proven to be effective for optimizing the cross-section of the BLS by thermal magnons, which is very sensitive to phase relations between numerous optical reflections in a multilayer system ensuring their constructive interferences.
